# Possible underreporting of pathogenic variants in *RAI1* causing Smith–Magenis syndrome

**DOI:** 10.1002/ajmg.a.62380

**Published:** 2021-06-04

**Authors:** Erik Boot, Cathelijne C. Linders, Sterre H. Tromp, Marie‐José van den Boogaard, Agnies M. van Eeghen

**Affiliations:** ^1^ Advisium, 's Heeren Loo Amersfoort The Netherlands; ^2^ The Dalglish Family 22q Clinic University Health Network Toronto Ontario Canada; ^3^ Department of Psychiatry & Neuropsychology Maastricht University Maastricht The Netherlands; ^4^ Department of Genetics University Medical Center Utrecht Utrecht The Netherlands; ^5^ Department of Pediatrics Emma Children's Hospital, Amsterdam University Medical Center Amsterdam The Netherlands

To the Editor,

Smith–Magenis syndrome (SMS; OMIM #182290, *607642) is a rare genetic neurodevelopmental disorder, estimated to affect 1:15,000–25,000 live births (Elsea & Girirajan, 2008; Greenberg et al., 1991). In the absence of prevalence studies, this estimate is however very approximate, and is thought to be closer to 1:15,000 due to underdiagnosis of the condition (Elsea & Girirajan, 2008). SMS is associated with multiple manifestations including congenital malformations (mainly of the heart and kidneys), intellectual disability, severe sleep disturbances, behavioral problems such as self‐injurious and aggressive behaviors, hypercholesterolemia, and overweight and obesity of variable severity (Elsea & Girirajan, 2008).

SMS is caused by a 17p11.2 deletion or a pathogenic variant in the retinoic acid‐induced gene 1 (*RAI1*) located within this chromosomal region (Slager et al., 2003). Previous studies have reported that approximately 90% of the patients have a 17p11.2 deletion. Of these, ~70% have a large and common deletion of 3.7 Mb, with the remaining 30% showing smaller or larger deletions ranging from 1.5 to 9 Mb (Edelman et al., 2007; Elsea & Girirajan, 2008; Finucane et al., 2021). Most of the SMS manifestations are thought to be the result of *RAI1* haploinsufficiency and 10% of the patients with SMS are reported to have a pathogenic variant within *RAI1* and no 17p11.2 deletion. Here, based on findings in a large SMS cohort, we propose that pathogenic variants in *RAI1* causing SMS may be underreported.

We reviewed available medical records from patients with a molecular diagnosis of SMS who visited the Dutch clinic for patients with SMS at 's Heeren Loo between 2002 and 2021. Originally a monodisciplinary medical clinic focusing on the treatment of sleep disorders (Spruyt et al., 2016), it has evolved to a multidisciplinary expert center providing patient‐centered care to patients and their families by health‐care experts from many specialties, including but not limited to intellectual disability medicine, psychology, speech–language pathology, dietetics and nutrition, and sensory integration therapy. We recorded information on ascertainment, demographic variables, and genetics, including age at last assessment, sex, age at genetic confirmation of the diagnosis, and details of the 17p11.2 deletion or *RAI1* variant when available. A waiver for formal approval was obtained from the Institutional Review Board of Amsterdam UMC, the Netherlands (#W20_098). To determine differences in molecular diagnostic age and sex between patients with a 17p11.2 deletion and patients with a *RAI1* variant, we used Mann–Whitney *U* and Fisher's exact tests, respectively. These analyses were two‐tailed, with statistical significance defined as *p* < 0.05, using IBM SPSS software (Statistics 25; SPSS, Inc, Chicago, IL).

Patients were referred to our clinic through four main sources, from most to least frequent: pediatrics, family medicine, medical genetics, and intellectual disability medicine. The sample comprised 87 patients with SMS aged 0–45 years (41 females, 47%) at last assessment. Sixty‐seven patients (77%) had a 17p11.2 deletion, of whom in 30 (45%) the deletion size was known: 23 (77%) had a common deletion of ~3.7 Mb, 5 (17%) a smaller, and 2 (7%) a larger deletion. Two patients (2%), both with a 17p11.2 deletion, had an additional genetic finding of clinical relevance: myotonic dystrophy type 1 and compound heterozygous variants in the phenylalanine hydroxylase gene associated with a mild phenylketonuria phenotype, respectively. Twenty patients (23%) had a pathogenic *RAI1* variant: 15 (83%) a frameshift and 3 (17%) a nonsense variant. In two patients, details about the variant were unknown. There were no statistically significant differences in sex between 17p11.2 patients (29 females; 43%) and those with a *RAI1* variant (12 females, 60%, *p* = 0.21). The median age at genetic confirmation of the diagnosis was statistically significant higher in the patients with a *RAI1* variant (11.0, range 2–34 years) compared to 17p11.2 patients (4.0, range 0–44 years, *p* = 0.000).

The data in our cohort suggest that the proportion of *RAI1* variants causing SMS is much higher than reported in previous studies; 23% compared to 10% of all patients with SMS (Elsea & Girirajan, 2008; Finucane et al., 2021). Pathogenic *RAI1* variants are likely to be underreported in previous reports, also resulting in an underestimation of the total prevalence of SMS.

A potential explanation for these findings is that clinicians may not always have suspicion of a genetic disorder in patients with *RAI1* variants because physical congenital anomalies such as cardiovascular and renal anomalies, chronic ear infections, hearing loss, speech and motor delay, and hypotonia are not common in these patients like in those with a 17p11.2 deletion (Girirajan et al., 2006). This may have been particularly an issue at the time of the initial studies on *RAI1* variants that were specifically based on individuals with the “typical SMS‐phenotype” but were not carriers of the deletion, whereas those not resembling “classical SMS” were not tested. One could also speculate about other factors that could contribute to the finding. For example, the clinical implementation of next‐generation sequencing (NGS) technologies has only been introduced in the last decade, now detecting patients with *RAI1* variants and no clinical suspicion of SMS, who would have otherwise been missed with traditional technology (Durmaz et al., 2015; Savatt & Myers, 2021). It is important to realize that *RAI1* haploinsufficiency as cause for SMS was only identified in 2003 (Slager et al., 2003).

The median age at genetic diagnosis in patients with a *RAI1* variant in our cohort was >10 years, and much higher than in patients with a 17p11.2 deletion. This suggests a diagnostic odyssey faced by patients with SMS and their families, especially in those with a *RAI1* variant, which may be prolonged in many patients due to underutilization of (modern) genomic diagnostic in routine clinical care (Savatt & Myers, 2021). Although we cannot rule that our findings may partially reflect ascertainment bias, it is plausible that still many patients with a pathogenic *RAI1* variant remain to be diagnosed.

Improved diagnostic genetic testing strategies, including NGS technology, for individuals with intellectual disability and/or multiple congenital anomalies in clinical practice will likely lead to a further increase of the number of patients diagnosed with pathogenic variants in *RAI1*. Clinically, this is important as the phenotype in *RAI1* patients may be different than in 17p11.2 patients with implications for genetic counseling and clinical decision making (Falco et al., 2017). For example, previous studies suggested that specific problem behaviors including self‐injurious behavior and overeating with overweight issues are more severe in patients with a *RAI1* variant, while congenital anomalies and short stature are probably limited to those with a 17p11.2 deletion (Edelman et al., 2007; Girirajan et al., 2006). Much is yet to be learned regarding the similarities and variable features for patients with 17p11.2 deletions and *RAI1* variants, given that the knowledge on genotype–phenotype correlations in *RAI1* patients are typically based on only a few patients and clinical information collected by proxy‐report (Edelman et al., 2007; Finucane et al., 2021; Vilboux et al., 2011). Future studies comparing phenotypic features of patients with 17p11.2 deletions to those with pathogenic *RAI1* variants may help uncover differences not yet fully appreciated.

## CONFLICT OF INTEREST

The authors declared no potential conflicts of interest.

## AUTHOR CONTRIBUTIONS

All persons who meet authorship criteria are listed as authors, and all authors certify that they have participated sufficiently in the work to take public responsibility for the content, including participation in the concept, design, analysis, interpretation of data, writing, or revision of the manuscript. Furthermore, each author certifies that this material or similar material has not been and will not be submitted to or published in any other publication before its appearance in the American Journal of Medical Genetics. Concept and design: E. Boot. Data collection: E. Boot, C. Linders, S. Tromp. Analysis and/or interpretation of data: E. Boot, M.J. van den Boogaard. Drafting the manuscript E. Boot. Reviewing the manuscript critically for important intellectual content: C. Linders, S. Tromp, M.J. van den Boogaard, A. van Eeghen. Approval of the version of the manuscript to be published: E. Boot, C. Linders, S. Tromp, M.J. van den Boogaard, A. van Eeghen.

## Data Availability

The study data that support our findings will be made available to qualified investigators upon reasonable request.

## References

[ajmga62380-bib-0001] Durmaz, A. A. , Karaca, E. , Demkow, U. , Toruner, G. , Schoumans, J. , & Cogulu, O. (2015). Evolution of genetic techniques: Past, present, and beyond. BioMed Research International, 2015, 461524. 10.1155/2015/461524 25874212 PMC4385642

[ajmga62380-bib-0002] Edelman, E. A. , Girirajan, S. , Finucane, B. , Patel, P. I. , Lupski, J. R. , Smith, A. C. , & Elsea, S. H. (2007). Gender, genotype, and phenotype differences in Smith‐Magenis syndrome: A meta‐analysis of 105 cases. Clinical Genetics, 71, 540–550. 10.1111/j.1399-0004.2007.00815.x 17539903

[ajmga62380-bib-0003] Elsea, S. H. , & Girirajan, S. (2008). Smith‐Magenis syndrome. European Journal of Human Genetics, 16, 412–421. 10.1038/sj.ejhg.5202009 18231123

[ajmga62380-bib-0004] Falco, M. , Amabile, S. , & Acquaviva, F. (2017). *RAI1* gene mutations: Mechanisms of Smith‐Magenis syndrome. The Application of Clinical Genetics, 10, 85–94. 10.2147/TACG.S128455 29138588 PMC5680963

[ajmga62380-bib-0005] Finucane, B. , Savatt, J. M. , Shimelis, H. , Girirajan, S. , & Myers, S. M. (2021). Birt‐Hogg‐Dubé symptoms in Smith‐Magenis syndrome include pediatric‐onset pneumothorax. American Journal of Medical Genetics. Part A, 185, 1922–1924. 10.1002/ajmg.a.62159 33666332 PMC9540435

[ajmga62380-bib-0006] Girirajan, S. , Vlangos, C. N. , Szomju, B. B. , Edelman, E. , Trevors, C. D. , Dupuis, L. , Nezarati, M. , Bunyan, D. J. , & Elsea, S. H. (2006). Genotype‐phenotype correlation in Smith‐Magenis syndrome: Evidence that multiple genes in 17p11.2 contribute to the clinical spectrum. Genetics in Medicine, 8, 417–427. 10.1097/01.gim.0000228215.32110.89 16845274

[ajmga62380-bib-0007] Greenberg, F. , Guzzetta, V. , Montes de Oca‐Luna, R. , Magenis, R. E. , Smith, A. C. , Richter, S. F. , Kondo, I. , Dobyns, W. B. , Patel, P. I. , & Lupski, J. R. (1991). Molecular analysis of the Smith‐Magenis syndrome: A possible contiguous‐gene syndrome associated with del(17)(p11.2). American Journal of Human Genetics, 49, 1207–1218. 1746552 PMC1686451

[ajmga62380-bib-0008] Savatt, J. M. , & Myers, S. M. (2021). Genetic testing in neurodevelopmental disorders. Frontiers in Pediatrics, 9, 526779. 10.3389/fped.2021.526779 33681094 PMC7933797

[ajmga62380-bib-0009] Slager, R. E. , Newton, T. L. , Vlangos, C. N. , Finucane, B. , & Elsea, S. H. (2003). Mutations in *RAI1* associated with Smith‐Magenis syndrome. Nature Genetics, 33, 466–468. 10.1038/ng1126 12652298

[ajmga62380-bib-0010] Spruyt, K. , Braam, W. , Smits, M. , & Curfs, L. M. (2016). Sleep complaints and the 24‐h melatonin level in individuals with Smith‐Magenis syndrome: Assessment for effective intervention. CNS Neuroscience & Therapeutics, 22, 928–935. 10.1111/cns.12653 27743421 PMC6492880

[ajmga62380-bib-0011] Vilboux, T. , Ciccone, C. , Blancato, J. K. , Cox, G. F. , Deshpande, C. , Introne, W. J. , Gahl, W. A. , Smith, A. C. M. , & Huizing, M. (2011). Molecular analysis of the *Retinoic Acid Induced 1* gene (*RAI1*) in patients with suspected Smith‐Magenis syndrome without the 17p11.2 deletion. PLoS One, 6, e22861. 10.1371/journal.pone.0022861 21857958 PMC3152558

